# Nigral overexpression of alpha-synuclein in the absence of parkin enhances alpha-synuclein phosphorylation but does not modulate dopaminergic neurodegeneration

**DOI:** 10.1186/s13024-015-0017-8

**Published:** 2015-06-23

**Authors:** Anne-Sophie Van Rompuy, Marusela Oliveras-Salvá, Anke Van der Perren, Olga Corti, Chris Van den Haute, Veerle Baekelandt

**Affiliations:** 1 Laboratory for Neurobiology and Gene Therapy, Department of Neurosciences, KU Leuven, Flanders, Belgium; 2 Inserm, U 975, CRICM, Hôpital de la Pitié-Salpêtrière, F-75013 Paris, France; 3 UPMC Université Paris 06, UMR_S975, F-75013 Paris, France; 4 CNRS, UMR 7225, F-75013 Paris, France; 5 Leuven Viral Vector Core, KU Leuven, 3000 Leuven, Belgium

**Keywords:** Parkinson’s disease, Alpha-synuclein, Alpha-synuclein phosphorylation, Parkin, Adeno-associated viral vectors, Knockout

## Abstract

**Background:**

Alpha-synuclein is a key protein in the pathogenesis of Parkinson’s disease. Mutations in the parkin gene are the most common cause of early-onset autosomal recessive Parkinson’s disease, probably through a loss-of-function mechanism. However, the molecular mechanism by which loss of parkin function leads to the development of the disease and the role of alpha-synuclein in parkin-associated Parkinson’s disease is still not elucidated. Conflicting results were reported about the effect of the absence of parkin on alpha-synuclein-mediated neurotoxicity using a transgenic approach. In this study, we investigated the effect of loss of parkin on alpha-synuclein neuropathology and toxicity in adult rodent brain using viral vectors. Therefore, we overexpressed human wild type alpha-synuclein in the substantia nigra of parkin knockout and wild type mice using two different doses of recombinant adeno-associated viral vectors.

**Results:**

No difference was observed in nigral dopaminergic cell loss between the parkin knockout mice and wild type mice up to 16 weeks after viral vector injection. However, the level of alpha-synuclein phosphorylated at serine residue 129 in the substantia nigra was significantly increased in the parkin knockout mice compared to the wild type mice while the total expression level of alpha-synuclein was similar in both groups. The increased alpha-synuclein phosphorylation was confirmed in a parkin knockdown cell line.

**Conclusions:**

These findings support a functional relationship between parkin and alpha-synuclein phosphorylation in rodent brain.

## Background

Parkinson’s disease (PD) is the second most common neurodegenerative disorder. Neuropathologically, it is characterized by the progressive loss of dopaminergic neurons in the substantia nigra (SN) and the presence of proteinaceous intracellular inclusions called Lewy bodies (LBs) and Lewy neurites in the surviving neurons [1]. Although the etiology of sporadic PD remains still unclear, the discovery of genes linked to familial forms of the disease has improved our understanding of the pathogenic mechanisms leading to PD.

Point mutations and multiplications of the α-synuclein (α-SYN) gene, SNCA, cause a rare familial autosomal dominant form of PD [2]. α-SYN is a small protein of 140 amino acids that is widely expressed in the brain and localizes predominantly to presynaptic terminals [3]. The biological function of α-SYN remains unknown, although it’s involvement in dopamine transmission and biosynthesis [4,5], synaptic plasticity [6] and turnover of synaptic vesicles has been suggested [7]. Under pathological conditions, including mutations and increased expression levels, α-SYN has the propensity to adopt a β-sheet rich conformation which leads to the formation of oligomers and fibrillar aggregates [8]. This fibrillar form of α-SYN is the main protein component of LBs and Lewy neurites, indicating that α-SYN plays a crucial role in the pathogenesis of PD [9]. Moreover, animal models based on overexpression of wild type (WT) or mutant α-SYN, recapitulate some of the main hallmarks of PD, including neurodegeneration, motor dysfunction and inclusion formation [10].

Mutations in the parkin gene are the major cause of early-onset autosomal recessive PD [11]. Parkin has been identified as an E3 ubiquitin-ligating enzyme, catalyzing the attachment of ubiquitin to substrate proteins, which consequently leads to their proteasomal degradation [12-14]. It was therefore suggested that the loss of parkin function due to disease-causing mutations might damage neurons through the accumulation of toxic proteins. On the other hand, evidence is accumulating that parkin ubiquitin ligase activity also contributes to non-degradative poly- and mono-ubiquitination, which are involved in alternative cellular pathways, including mitochondrial quality control [15] and signal transduction cascade activation [16,17]. However, the molecular mechanism by which loss of parkin function leads to the development of PD is still not completely elucidated. More specifically, the role of α-SYN in parkin-associated PD remains unclear, although there are lines of evidence suggesting a potential link between parkin and α-SYN. On one hand it has been reported that the unmodified form of α-SYN does not interact with parkin [18] and no accumulation of α-SYN was detected in the brain of parkin knockout (parkin^−/−^) mice [19-21]. On the other hand, in about two thirds of the seventeen neuropathological reports of patients with parkin mutations published to date, no LBs were found, indicating that parkin might play a role in LB formation[22]. Furthermore, overexpression of parkin provides protection against α-SYN toxicity in a variety of cellular and animal models [23-29]. Conflicting results were published in a number of studies using a transgenic approach to investigate the effect of parkin on α-SYN-induced neurotoxicity. The neurodegenerative phenotype was unexpectedly delayed in the absence of parkin in A30P α-SYN transgenic mice [30], while no effect of loss of parkin on neuropathology was found in A53T α-SYN transgenic mice [31]. In a third publication, an increase in damaged mitochondria in neurons of the SN and a reduction of complex I activity was reported in old double mutant mice generated by the crossing of parkin^−/−^ mice with double mutated A53T and A30P α-SYN overexpressing mice [32]. However, these results in transgenic mice showed that the absence of parkin did not clearly affect α-SYN-induced neurodegeneration, except for some minor changes.

In the present study, we chose an alternative approach to investigate the effect of the absence of parkin on α-SYN-induced cell death in adult rodent brain using viral vector technology. Therefore, we overexpressed human WT α-SYN in the SN of parkin^−/−^ and wild type (parkin^+/+^) mice with recombinant adeno-associated viral (rAAV) vectors. Subsequently, we analyzed the degree of neurodegeneration and synucleinopathy in the SN of those mice.

## Results

### Absence of parkin does not increase the sensitivity to dopaminergic degeneration induced by a high dose of rAAV2/7-WT α-SYN

In a previous study, we showed that rAAV2/7-mediated overexpression of WT α-SYN in the SN of mice resulted in a dose-dependent, progressive dopaminergic neurodegeneration [33]. Therefore, to study the effect of the absence of parkin on α-SYN induced dopaminergic cell death, in a first experiment we stereotactically injected a rAAV2/7 vector encoding WT α-SYN in the right SN of adult parkin^−/−^ and parkin^+/+^ mice at a vector titer of 4E + 11 genome copies (GC)/ml (high vector titer, see experimental design in Table 1).The animals were analyzed at 1 week and 4 weeks after injection. As additional control, a group of parkin^−/−^ and parkin ^+/+^ mice was injected with a similar titer of rAAV2/7 vector coding for enhanced green fluorescent protein (eGFP).

**Table 1:** **Experimental design**

	**Group**					
**Analysis time point (Immunohistochemistry)**	**rAAV2/7-WT** **α** **SYN**		**rAAV2/7-eGFP**		**rAAV2/7-WT** **α** **SYN**	
	**(4E + 11 GC/ml)**		**(4E + 11 GC/ml)**		**(1E + 11 GC/ml)**	
	**high** **α** **-SYN dose**				**low** **α** **-SYN dose**	
	**parkin** ^**+/+**^	**parkin** ^**−/−**^	**parkin** ^**+/+**^	**parkin** ^**−/−**^	**parkin** ^**+/+**^	**parkin** ^**−/−**^
1 week	n = 4	n = 4			n = 2	n = 3
4 weeks	n = 15	n = 16	n = 5	n = 6	n = 10	n = 13
8 weeks					n = 6	n = 5
16 weeks					n = 6	n = 6

Adult 2 to 4-month-old parkin^+/+^ and parkin^−/−^ mice were stereotactically injected in the right SN with 2 μl of rAAV2/7-WT α-SYN vector at a titer of 4E + 11 GC/ml (high α-SYN dose) or 1E + 11 GC/ml (low α-SYN dose) or rAAV2/7-eGFP vector at a titer of 4E + 11 GC/ml. At the mentioned time points after injection, animals were perfused for immunohistochemical analysis.

**Figure 1:**
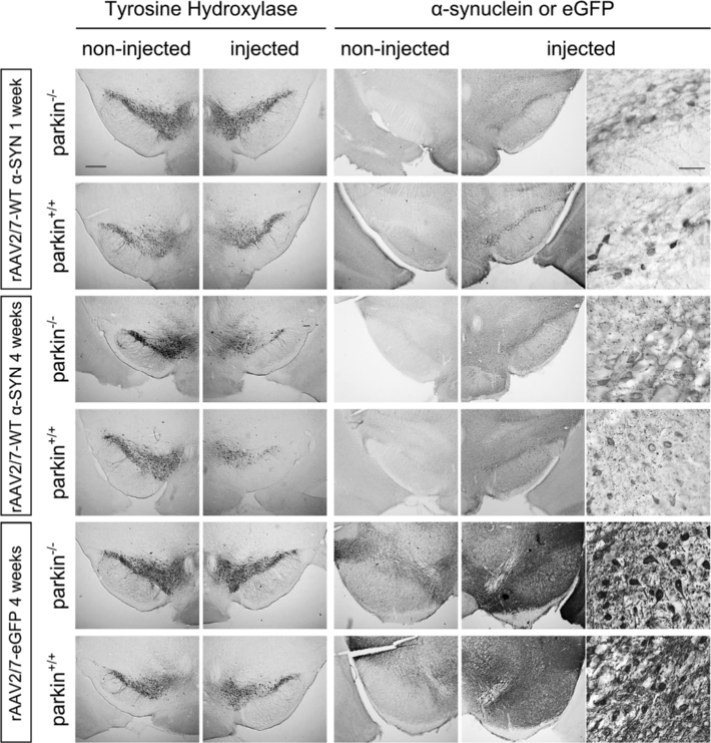
Overexpression of α-SYN or eGFP in mouse SN with high titer rAAV2/7 vectors. Immunohistochemical staining for TH (left panels) shows cell loss in the injected side of both the parkin^−/−^ and the parkin^+/+^ mice at 4 weeks after injection with a high titer of rAAV2/7-WT α-SYN. Immunohistochemical stainings for α-SYN and eGFP (middle and right panels) show wide expression of these proteins in the injected side of the SN. Right panels are magnifications of the adjacent left panel. Scale bars overviews = 400 μm and magnifications = 50 μm.

**Figure 2:**
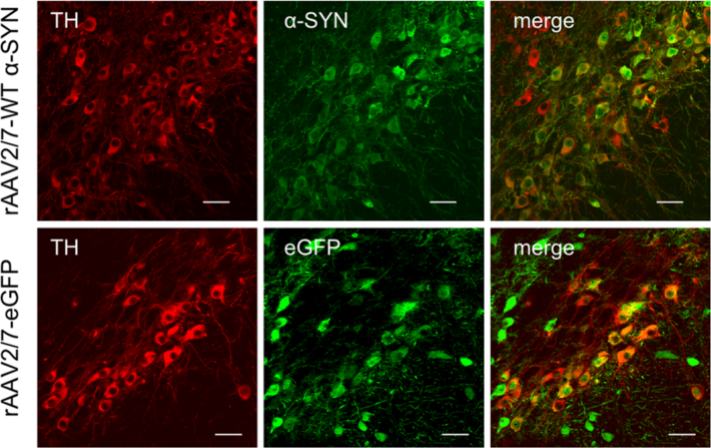
High transduction efficiency of mouse dopaminergic neurons with rAAV2/7-WT α-SYN and rAAV2/7-eGFP. Fluorescent double stainings for TH and α-SYN at 1 week or TH and eGFP at 4 weeks after injection of rAAV2/7-WT α-SYN or rAAV2/7-eGFP respectively, demonstrate that the majority of the dopaminergic neurons of the injected side of the SN is transduced. Scale bar = 25 μm.

**Figure 3:**
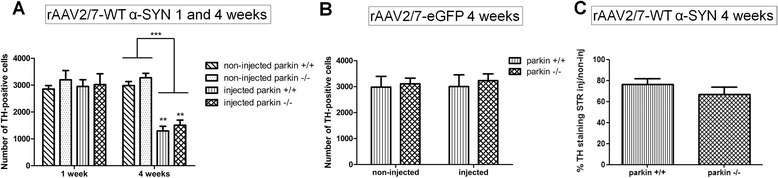
A high dose of rAAV2/7-WT α-SYN induces similar dopaminergic degeneration in parkin^−/−^ and parkin^+/+^ mice. Stereological quantification of the number of TH-positive neurons in the SN of parkin^+/+^ and parkin^−/−^ mice at **(A)** 1 week and 4 weeks after injection with a high titer of rAAV2/7-WT α-SYN and at **(B)** 4 weeks after injection with rAAV2/7-eGFP. **(C)** quantification of TH staining in striatum (injected over non-injected side) of parkin^+/+^ and parkin^−/−^ mice 4 weeks after injection. Asterisks depict significant decrease respective to 1 week, unless specified otherwise. (Mean ± SEM, two-way ANOVA followed by Bonferroni post-hoc test, ** p < 0.01 ***p < 0.001, 1 week rAAV2/7-WT α-SYN: n = 4; 4 weeks rAAV2/7-WT α-SYN: n = 15-16; 4 weeks rAAV2/7-eGFP: n = 5-6).

Immunohistochemical stainings for α-SYN and eGFP revealed high transgene expression in the SN for both vectors (Figure 1). With confocal analysis we observed a transduction efficiency of the dopaminergic neurons of approximately 85% (Figure 2). To investigate the degree of dopaminergic neurodegeneration, we stereologically quantified the number of tyrosine hydroxylase (TH)-positive cells in the SN. At 4 weeks after injection, the rAAV2/7-WT α-SYN induced a dopaminergic lesion of 59 ± 6% compared to the non-injected side in the parkin^+/+^ mice, which is in agreement with our previous study [33] (Figure 1 and 3A). In the parkin^−/−^ mice a comparable dopaminergic cell loss of 52 ± 6% was observed. The rAAV2/7-eGFP injected mice did not show any loss of TH-positive cells (Figure 1 and 3B). The loss of dopaminergic terminals in the striatum was comparable between parkin^+/+^ (24 ± 5.2%) and parkin^−/−^ (33 ± 6.9%) mice (Figure 3C). Thus, we conclude that the sensitivity of parkin^−/−^ mice and parkin^+/+^ mice to dopaminergic degeneration induced by a high dose of rAAV2/7-WT α-SYN is similar.

### Overexpression of α-SYN with rAAV2/7 increases phosphorylation of α-SYN at serine residue 129 in parkin^−/−^ mice compared to parkin^+/+^ mice

**Figure 4:**
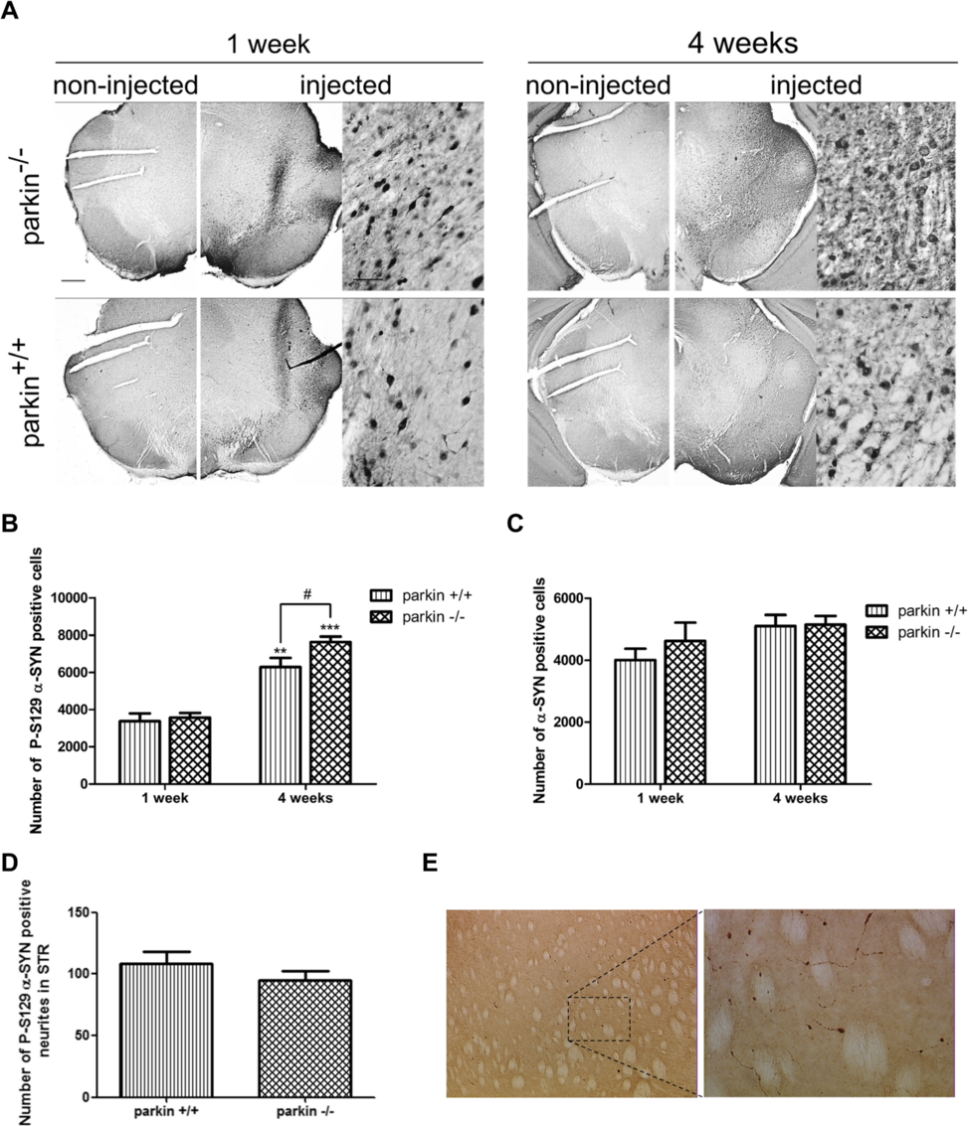
Increased phosphorylation of α-SYN at S129 in parkin^−/−^ mice compared to parkin^+/+^ mice. **(A)** Representative images of P-S129 α-SYN expression in the SN of parkin^−/−^ and parkin^+/+^ mice at 1 week and 4 weeks after injection with a high titer of rAAV2/7-WT α-SYN. Right panels are magnifications of the overviews of the injected side (middle panels). Scale bar overviews = 400 μm and magnifications = 50 μm. **(B)** Stereological quantification of the number of P-S129 α-SYN positive cells in the injected side of the SN of parkin^+/+^ and parkin^−/−^ mice at 1 week (n = 4) and 4 weeks (n = 15-16) after injection with a high titer of rAAV2/7-WT α-SYN. (Mean ± SEM, two-way ANOVA followed by Bonferroni post-hoc test, # p < 0.05 versus parkin^+/+^, **p < 0.01 versus 1 week, ***p < 0.001 versus 1 week). **(C)** Stereological quantification of the number of α-SYN positive cells in the injected side of the SN of parkin^+/+^ and parkin^−/−^ mice at 1 week (n = 4) and 4 weeks (n = 15-16) after injection with a high titer of rAAV2/7-WT α-SYN. (Mean ± SEM). **(D)** Quantification of the number of P-S129 α-SYN positive neuritic inclusions in the striatum at 4 weeks (n = 14–15, Mean ± SEM). **(E)** Picture of the P-S129 α-SYN staining in the striatum (left: 10 x) and magnification (right: 40 x) of neuritic inclusions. Scale bar = 50 μm.

In a next step, we determined the number of cells in the SN that were positive for α-SYN phosphorylated at serine residue 129 (P-S129), a form of α-SYN which is considered to be the pathological form of α-SYN and the most abundant modification of α-SYN in LBs [34,35]. Therefore, we performed an immunohistochemical staining with an antibody specifically recognizing this phosphorylated form of α-SYN [34] and stereologically quantified the number of positive cells in the injected side of the whole SN. No P-S129 positive cells were observed in the non-injected side of the SN, indicating that only phosphorylation of the overexpressed human α-SYN is within the limits of detection. At 1 week after injection, no difference was observed between parkin^−/−^ mice (3563 ± 259 cells) and parkin^+/+^ mice (3371 ± 416 cells). At 4 weeks after injection, the number of cells positive for P-S129 α-SYN was increased in both groups when compared to 1 week. Interestingly, this number was significantly higher in the parkin^−/−^ mice (7632 ± 291 cells) than in the parkin^+/+^ mice (6288 ± 495 cells) (p = 0,027) (Figure 4A-B). This higher number of P-S129 α-SYN positive cells in the parkin^−/−^ mice was not caused by higher expression levels of α-SYN, since the total number of α-SYN positive cells in the SN was similar in the parkin^+/+^ and parkin^−/−^ mice at both time points (Figure 4C). Differences in the affinities of the P-S129 α-SYN antibody and α-SYN antibody explain the apparent lower number of α-SYN positive cells compared to the number of P-S129 α-SYN positive cells. We also stained the striatum for P-S129 α-SYN to check if the dopaminergic terminals also contain phosphorylated α-SYN. We detected P-S129 α-SYN-positive neuritic inclusions (Figure 4E) but no difference in the number of these inclusions was observed between parkin^+/+^ and parkin^−/−^ mice at 4 weeks after injection (Figure 4D).

### A low dose of rAAV2/7-WT α-SYN induces slower but similar dopaminergic degeneration and increased α-SYN phosphorylation in parkin^−/−^ mice

The enhanced phosphorylation of α-SYN in the parkin^−/−^ mice observed in the previous experiment was not paralleled by an increase in dopaminergic cell death. We reasoned that this might be due to the very fast and robust degenerative process, precluding the detection of subtle differences. Therefore, we decided to repeat the experiment with a 4 times lower dose of rAAV2/7-WT α-SYN (1E + 11 GC/ml, low vector titer). In this experiment, animals were analyzed at 1 week, 4 weeks, 8 weeks and 16 weeks after injection (see experimental design in Table 1).

**Figure 5:**
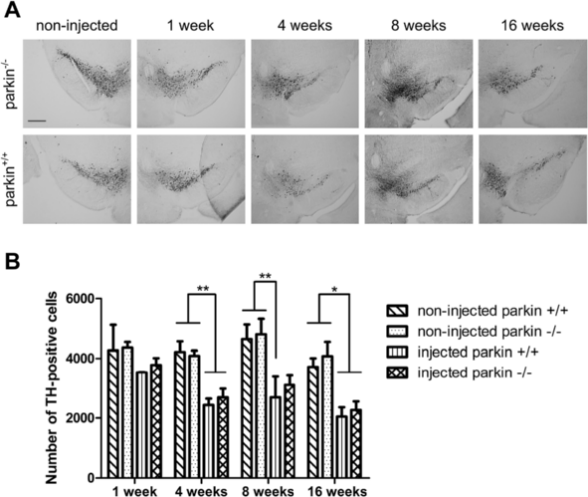
A low dose of rAAV2/7-WT α-SYN induces similar dopaminergic degeneration in parkin^−/−^ and parkin^+/+^ mice. **(A)** Representative images of immunohistochemical staining for TH in the SN at 1 week, 4 weeks, 8 weeks and 16 weeks after injection with a low titer of rAAV2/7-WT α-SYN. Scale bar = 400 μm. **(B)** Stereological quantification of the number of TH-positive neurons in the SN of parkin^+/+^ and parkin^−/−^ mice at 1 week, 4 weeks, 8 weeks and 16 weeks after injection with a low titer of rAAV2/7-WT α-SYN**.** (Mean ± SEM, two-way ANOVA followed by Bonferroni post-hoc test, *p < 0.05, **p < 0.01, n = 2-3 at 1 week, n = 10-13 at 4 weeks, n = 5-6 at 8 weeks, n = 6 at 16 weeks).

As expected, stereological quantification of the number of surviving dopaminergic neurons revealed a milder dopaminergic cell loss at 4 weeks (approximately 40%) compared to the high titer of rAAV2/7-WT α-SYN. At 8 weeks and 16 weeks, in both groups the dopaminergic degeneration was not further progressive, suggesting that with the low titer of rAAV2/7-WT α-SYN the maximum amount of degeneration was already reached at 4 weeks after injection. However, the TH-positive cell loss was again comparable between the parkin^−/−^ and parkin^+/+^ mice at all time points (e.g. at 4 weeks 35 ± 7% and 41 ± 5% TH-positive cell loss, respectively compared to the non-injected side) (Figure 5A-B). These results confirm that the absence of parkin does not alter the susceptibility to α-SYN induced dopaminergic cell death.

**Figure 6:**
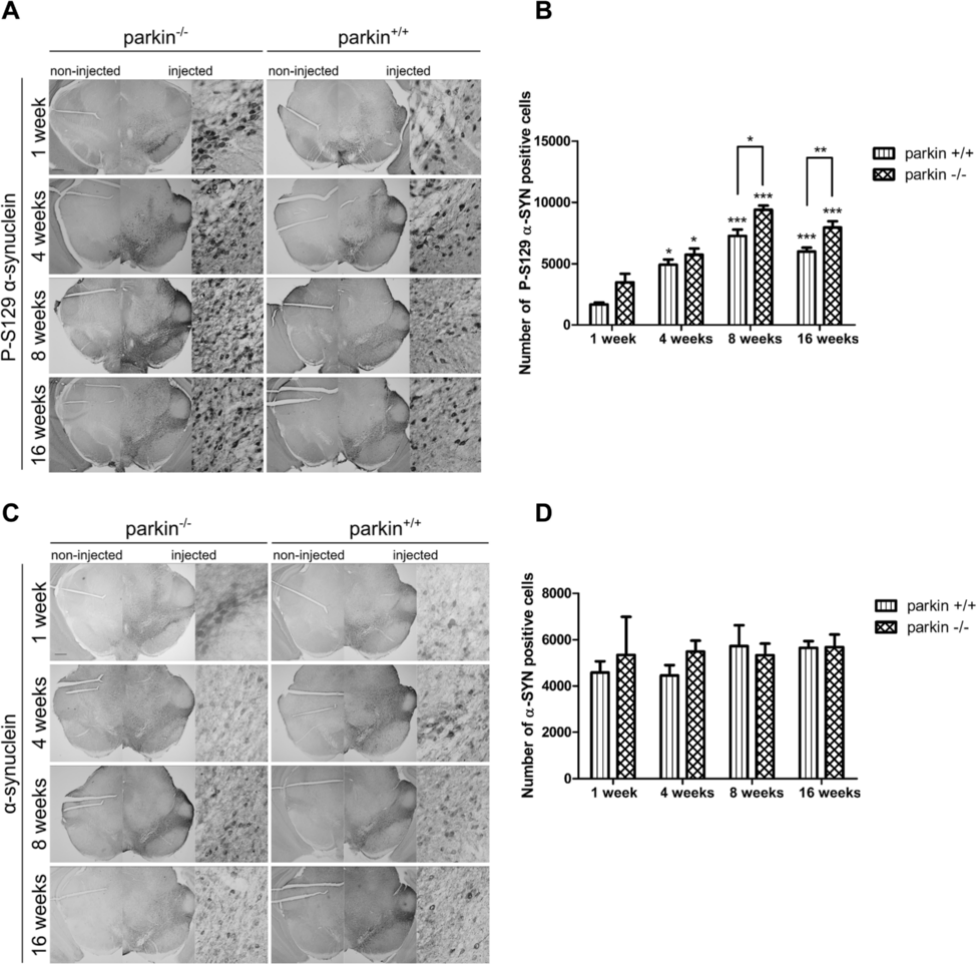
Increased P-S129 α-SYN in parkin^−/−^ mice after injection with a low titer of rAAV2/7-WT α-SYN. **(A)** Representative images of P-S129 α-SYN expression in the SN of parkin^−/−^ and parkin^+/+^ mice at 1 week, 4 weeks, 8 weeks and 16 weeks after injection with a low titer of rAAV2/7-WT α-SYN. Right panels are magnifications of the overview of the injected side (middle panels). Scale bar overviews = 400 μm and magnifications = 50 μm. **(B)** Stereological quantification of the number of P-S129 α-SYN positive cells in the injected side of the SN of parkin^+/+^ and parkin^−/−^ mice at 1 week (n = 2-3), 4 weeks (n = 10-13), 8 weeks (n = 5-6) and 16 weeks (n = 6) after injection. Asterisks depict significant increase respective to 1 week, unless specified otherwise. (Mean ± SEM, two-way ANOVA followed by Bonferroni post-hoc test, student’s *T*-test followed by Benjamini-Hochberg to compare parkin^+/+^ and parkin^−/−^ separately at the different time points, *p < 0.05, **p < 0.01, ***p < 0.001). **(C)** Representative images of α-SYN expression in the SN of parkin^−/−^ and parkin^+/+^ mice at 1 week, 4 weeks, 8 weeks and 16 weeks after injection with a low titer of rAAV2/7-WT α-SYN. Right panels are magnifications of the overview of the injected side (middle panels). Scale bar overviews = 400 μm and magnifications = 50 μm. **(D)** Stereological quantification of the number of α-SYN positive cells in the injected side of the SN of parkin^+/+^ and parkin^−/−^ mice at 1 week (n = 2-3), 4 weeks (n = 10-13), 8 weeks (n = 5-6) and 16 weeks (n = 6) after injection with a low titer of rAAV2/7-WT α-SYN. (Mean ± SEM).

We also investigated the effect of absence of parkin on α-SYN phosphorylation in the low dose α-SYN set-up. The number of P-S129 α-SYN positive cells in the injected side progressively increased over time until 8 weeks and remained stable at 16 weeks after viral vector delivery to the SN (Figure 6A-B). Here again, we observed a significantly higher level of α-SYN phosphorylation in the parkin^−/−^ mice compared to the parkin^+/+^ mice at 8 weeks and 16 weeks after injection (respectively 9389 ± 337 cells versus 7263 ± 532 cells at 8 weeks, p = 0,0179; 7954 ± 518 cells versus 6000 ± 313 cells at 16 weeks, p = 0,009). Immunohistochemical staining for α-SYN confirmed expression of α-SYN up to 16 weeks after injection (Figure 6C). As seen before, the total number of α-SYN positive cells was similar in the parkin^−/−^ mice and the parkin^+/+^ mice at the 4 different time points (Figure 6D).

**Figure 7:**
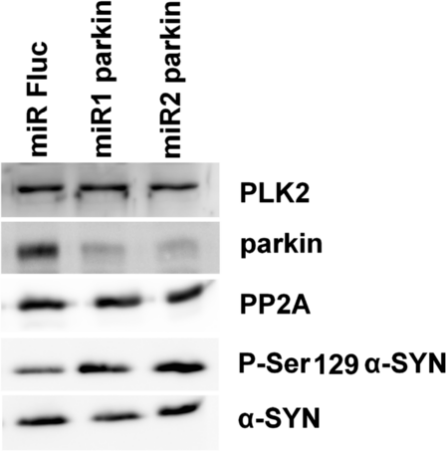
Increased P-S129 α-SYN in human SHSY5Y neuroblastoma cells after parkin knockdown. Western blotting against PLK2, parkin, PP2A, P-Ser129 α-SYN and α-SYN. The miRs against parkin induced both parkin knockdown and an increase in P-Ser129 α-SYN signal without affecting α-SYN, PLK2 and PP2A levels. A miR against firefly luciferase (Fluc) was used as control.

To investigate the mechanism behind this increased α-SYN phosphorylation in the absence of parkin we induced stable parkin knockdown using microRNA (miR)-based lentiviral vectors in human SHSY5Y neuroblastoma cells overexpressing WT human α-SYN. Two miRparkin lentiviral vectors induced efficient knockdown of parkin as shown by Q-RT-PCR (data not shown) and Western blotting (Figure 7). In agreement with the *in vivo* data we observed an increase of α-SYN phosphorylation at serine residue 129 without affecting the total α-SYN levels in cell culture (Figure 7). Since Polo-Like-Kinase-2 (PLK2) was shown to phosphorylate α-SYN at S129 [36] and protein phosphatase-2A (PP2A) is a known α-SYN phosphatase at S129 [37], we verified the levels of PLK2 and PP2A in both parkin knockdown and the control cell lines but no differences were observed  in expression of either protein (Figure 7).

We can conclude that overexpression of α-SYN in the absence of parkin enhances phosphorylation of α-SYN at serine residue 129 but does not affect the degree of dopaminergic neurodegeneration.

## Discussion

This study was designed to investigate the effect of the loss of parkin on α-SYN induced neurotoxicity in rodent brain. We found that the absence of parkin did not alter the vulnerability of dopaminergic neurons to WT α-SYN induced neurodegeneration. However, the number of P-S129 α-SYN positive cells in the SN of parkin^−/−^ mice was increased compared to parkin^+/+^ mice. This increase in the number of P-S129 α-SYN positive cells in the parkin^−/−^ mice was not due to differences in expression level of α-SYN, since the total number of α-SYN-positive cells was similar in both groups, These results were reproduced in a second, independent experiment performed with a 4 times lower titer of rAAV2/7-WT α-SYN.

A number of previous studies already addressed the question if the absence of parkin affects the development of α-synucleinopathy using a transgenic strategy. However, inconsistent results were reported, since in one study no effect was found in A53T α-SYN transgenic mice [31], whereas in a second study the loss of parkin unexpectedly mitigated the α-SYN phenotype in A30P α-SYN transgenic mice [30]. In the present study, we opted for an alternative approach with viral vector-mediated overexpression of WT α-SYN in the SN of parkin^−/−^ and parkin^+/+^ mice. rAAV vectors are an attractive tool for gene delivery in the brain, since they provide several advantages: specific brain regions can be targeted, the transduction efficiency in dopaminergic neurons is high, and a long-lasting and stable expression of the transgene at different doses can be achieved with a single delivery [38]. In addition, in a previous study in our own research group, we showed that rAAV2/7-mediated WT α-SYN overexpression in mouse SN leads to a dose-dependent, progressive dopaminergic cell death [33].

With our strategy, we did not observe a difference in sensitivity to WT α-SYN induced dopaminergic cell death between parkin^−/−^ and parkin^+/+^ mice. The strength of our study is that it allowed us to specifically investigate the sensitivity of the dopaminergic cell population to α-SYN toxicity in the absence of parkin. Since the majority of α-SYN transgenic mice do not develop a robust dopaminergic phenotype, they are less suitable to study the effect of parkin deficiency and α-synucleinopathy in nigral dopaminergic neurons [39,40]. On the other hand, our finding that loss of parkin does not exacerbate dopaminergic degeneration is partly consistent with the results in the transgenic mice, since in those studies, no difference in dopaminergic cell survival was found between the α-SYN overexpressing mice and the parkin^−/−^ - α-SYN double transgenic mice, indicating that the complete absence of parkin does not affect dopaminergic cell survival [30,31]. A possible explanation for this somewhat unexpected observation might be the presence of compensatory mechanisms in the parkin^−/−^ mice, which may counterbalance for the total loss of parkin protein occurring already during embryonic development. Reports of an increased sensitivity of striatal metabotropic glutamate receptors and elevated levels of reduced glutathione in parkin^−/−^ mice indicate that such compensatory adaptations exist [41,42]. Locoregional downregulation of parkin with viral vectors or the generation of conditional parkin knockout animals may be a valuable strategy to overcome this issue. Indeed, it was recently reported that adult depletion of parkin in the SN of conditional parkin^−/−^ mice resulted in a progressive loss of dopaminergic neurons up to 40%, a phenotype that has never been observed in the constitutive parkin^−/−^ mice [21,43].

The lack of increased vulnerability of parkin^−/−^ mice to WT α-SYN induced dopaminergic cell death was observed with two different doses of WT α-SYN. In the second experiment, we opted for a lower dose, because we reasoned that the dramatic dopaminergic cell loss achieved with the highest dose of WT α-SYN might hide small differences in sensitivity between parkin^−/−^ and parkin^+/+^ mice. Although the lower dose of WT α-SYN resulted in a milder dopaminergic cell death, the degree of degeneration was still considerable (approximately 40% at 4 weeks). Therefore, we cannot exclude that subtle differences in sensitivity might emerge when using even lower overexpression of α-SYN or at later time points than analyzed in this study.

In a next step, we wondered whether the absence of parkin would influence the phosphorylation of α-SYN at serine residue 129, since the level of P-S129 α-SYN is highly elevated in the brains of PD patients [34,35]. We found that the number of nigral cells positive for P-S129 α-SYN was significantly higher in the parkin^−/−^ mice compared to parkin^+/+^ mice. This was not the case for the dopaminergic terminals in the striatum. This discrepancy might suggest that mainly the non-dopaminergic neurons show an increased phosphorylation in the parkin^−/−^ mice or that the dopaminergic neurons with increased P-S129 α-SYN have relatively lower terminal densities. This increased phosphorylation is in apparent contradiction with the findings of Lo Bianco *et al.* who reported an increase in P-S129 α-SYN positive aggregates after overexpression of parkin together with A30P α-SYN in the rat SN using lentiviral vectors [25]. That observation was associated with a protective effect of parkin overexpression on A30P α-SYN induced dopaminergic degeneration. Furthermore, no increase in P-S129 α-SYN abundance was noticed in A30P α-SYN transgenic mice on a parkin^−/−^ background [30]. On the contrary, and in agreement with our results, lentiviral vector-mediated co-expression of parkin with WT α-SYN in the striatum of rats reduced the levels of P-S129 α-SYN [24]. A similar result was found in the striatum of macaque monkeys when parkin and WT α-SYN were overexpressed by means of rAAV vectors, although in this study a decrease in total α-SYN was also reported [29]. In the study with the A53T α-SYN transgenic mice crossed with parkin^−/−^ mice phosphorylation of α-SYN has not been investigated [31]. So far, we cannot clearly explain the inconsistencies between these reports, although the most obvious explanation is differences in experimental conditions. Altogether, the studies performed with WT α-SYN, including ours, point towards a correlation between decreased levels of parkin and increased phosphorylation of α-SYN at S129, whereas in the studies with mutant A30P α-SYN either the reverse or no effect is suggested. Thus, it is possible that the influence of parkin on S129-phosphorylation is different for WT α-SYN than for A30P α-SYN, since the two forms also differ in other properties including aggregation [44] and membrane-binding [45].

At this point, we can only speculate about the mechanism behind the increased phosphorylation of WT α-SYN in the absence of parkin. No direct binding of parkin to P-S129 α-SYN was observed in brain extracts of A30P α-SYN transgenic mice [30]. We also quantified the percentage of ubiquitin and α-SYN double-positive cells but no difference was seen between parkin^+/+^ and parkin^−/−^ mice (data not shown). This suggests that the increased phosphorylation of WT α-SYN is not a consequence of a difference in ubiquitination of α-SYN. It is possible that parkin modulates kinases and phosphatases regulating the phosphorylation of α-SYN at S129. In agreement with this hypothesis it was reported that overexpression of α-SYN in the brain of rats resulted in an increase in the level of Polo-Like-Kinase-2 (PLK2) and this increase was annihilated when parkin was co-expressed with α-SYN [24]. Absence of parkin might then result in a more pronounced increase in PLK2 levels and therefore increased S129 phosphorylation of α-SYN, since PLK2 is known to phosphorylate α-SYN at S129 [36]. We performed stainings for PLK2 on brain sections, but we failed to reliably detect endogenous expression levels of PLK2 (data not shown). Therefore we induced stable parkin knockdown in human SHSY5Y neuroblastoma cells overexpressing WT α-SYN. Interestingly, the increased S129 phosphorylation of α-SYN after parkin knockdown was replicated in this cell culture model, without effect on total α-SYN level. However, PLK2 protein levels were not altered after parkin knockdown in cell culture. Another potential mechanism involves protein phosphatase-2A (PP2A) that has been shown to dephosphorylate α-SYN at S129 [37]. Indeed, the level of protein phosphatase-2A (PP2A) was reportedly increased when parkin was co-expressed with α-SYN compared to expression of α-SYN alone in rat striatal extracts [24]. Absence of parkin might then decrease PP2A levels in these conditions, resulting in increased S129 phosphorylation of α-SYN. However, we failed to detect alterations in PP2A expression in the parkin knockdown cells. Thus, our data suggest that the mechanism behind the increased α-SYN phosphorylation might be independent from the PLK2 or PP2A pathway, although we cannot exclude changes in activity of either PLK2 or PP2A.

Furthermore, it has been demonstrated that 26S proteasomal activity is decreased in parkin^−/−^ mice and parkin null *Drosophila* [46]. Also proteasomal dysfunction and S129 phosphorylation of α-SYN have been linked before. First, two independent studies describe that proteasomal inhibition increases casein kinase 2 activity, another kinase mediating phosphorylation of α-SYN at S129 [47], resulting in enhanced S129 phosphorylation of α-SYN [48,49]. Second, it was demonstrated that inhibition of the proteasome pathway resulted in the accumulation of P-S129 α-SYN without alteration in the total levels of α-SYN, suggesting that P-S129 α-SYN specifically undergoes degradation by the proteasome pathway [50].

In the present study, the increased level of P-S129 α-SYN in the parkin^−/−^ mice was not associated with increased dopaminergic degeneration, which is intuitively in contradiction with the knowledge that ± 90% of α-SYN within LBs from PD patients is phosphorylated at S129 [34,35]. The toxicity of P-S129 α-SYN *in vivo* has been studied extensively in the last years using mutated forms of α-SYN in which S129 is replaced either with an aspartate (S129D), to mimic phosphorylation or with an alanine (S129A), to block phosphorylation. However, conflicting results were reported. Expression of S129D α-SYN in *Drosophila* resulted in an enhanced toxicity, whereas no dopaminergic cell loss was observed if S129A α-SYN was expressed [51]. On the contrary, *Caenorhabditis elegans* overexpressing S129A α-SYN showed severe motor dysfunction and synaptic abnormalities, unlike worms overexpressing S129D α-SYN that exhibited a nearly normal phenotype [52]. Two studies using rAAV vectors in rats found expression of S129A α-SYN to be more toxic than S129D α-SYN [53,54]. In a third study, there was no difference between the two forms of α-SYN [55]. In a recent study, S129D α-SYN expression in rat SN resulted in an accelerated striatal dopaminergic fiber loss and an earlier appearance of motor deficits compared to S129A α-SYN, although the nigral degeneration was similar [56]. However, the S129D mutant might not completely mimick the constitutively phophorylated α-SYN. In another study phosphorylation of A53T α-SYN was induced by rAAV-mediated overexpression of G-protein-coupled receptor kinase 6 in rats, which resulted in accelerated A53T α-SYN induced neurodegeneration [57], in contrast to our data. Species differences might be involved, since we observed that rats are more sensitive to rAAV-α-SYN-induced neurodegeneration compared to mice [33,58]. Altogether, the role of S129 phosphorylation of α-SYN in neurodegeneration still remains unclear.

## Conclusions

In the present study, we have shown that the vulnerability of mouse dopaminergic neurons to α-SYN toxicity is not altered in the absence of parkin, but that the loss of parkin enhances phosphorylation of α-SYN at S129. Additional studies will be required to elucidate the molecular mechanism behind our findings and the significance for the pathogenesis of parkin-associated PD.

## Methods

### Cloning of rAAV vector plasmids

The plasmids for the rAAV vector production were previously described [38,59]. These plasmids include the construct for the AAV2/7 serotype, the AAV transfer plasmid and the pAdvDeltaF6 adenoviral helper plasmid. The transfer plasmids encoded as transgene eGFP or human WT α-SYN. The transgenes were under the control of the CMVie-enhanced Synapsin1 promotor.

### Recombinant rAAV vector production and purification

Vector production and purification were performed as previously described [38]. Briefly, the triple transfection into HEK293T cells was carried out using linear polyethylenimine solution. Vector particles were harvested from the supernatant and concentrated using tangential flow filtration. The concentrated supernatant was further purified using a discontinuous iodixanol step gradient. The final sample was aliquoted and stored at −80°C. Characterization of the rAAV stocks included real-time quantitative PCR analysis for genomic copy determination (presented as GC/ml) and silver-stained sodium dodecyl sulfate-polyacrylamide gel electrophoresis analysis for vector purity.

### Animals and stereotactic neurosurgery

All animal experiments were carried out in accordance with the European Communities Council Directive of 24 November 1986 (86/609/EEC) and approved by the Bioethical Committee of the KU Leuven (Belgium).

The transgenic parkin^−/−^ mice carry a homozygous deletion of exon 3 [41]. Parkin^+/+^ mice in the whole first 'high α-SYN dose’ experiment (n = 19), including rAAV2/7-eGFP (n = 5), and part in the second ‘low α-SYN dose’ experiment (1 week all and 4 weeks: 6 out of 10) were age- and gender-matched C57BL/6 J mice (Janvier, Le-Genest-Saint-Isle, France) (see Table 1). Rest of parkin^+/+^ mice in the ‘low α-SYN dose’ experiment were age- and gender-matched wild type littermates. Parkin genotyping was performed as described in [30]. No differences were observed in number of TH-positive cells, Ser-129-P α-SYN positive cells and α-SYN positive cells between the C57Bl6 mice and wild type littermates at 4 weeks after injection with the low titer of rAAV2/7-WT α-SYN (data not shown). Therefore, we concluded that potential minor differences in background did not influence our outcome. The age of the mice was 2 months in the first experiment and varied between 2 and 4 months in the second experiment. The mice were housed under a normal 12-h light/dark cycle with free access to pelleted food and tap water. All surgical procedures were performed using aseptic techniques and ketamine (75 mg/kg IP, KETALAR®, Pfizer, Elsene, Belgium) and medetomidine (1 mg/kg, DORMITOR®, Pfizer) anesthesia. Following anesthesia the mice were placed in a stereotactic head frame (Stoelting Co, Wood Dale, IL, USA). The skull was exposed by a midline incision and a small hole was drilled in the appropriate location, using bregma as reference point. 2 μl of rAAV vector were injected at a rate of 0.25 μl/min with a 30-gauge needle on a 10 μl Hamilton syringe. Stereotactic coordinates used for the right mouse SN were anteroposterior −3.1 mm and medio-lateral −1.2 mm relative to bregma, and dorsoventral −4.0 mm, from the dura surface. The needle was left in place for an additional 5 min before being slowly retracted. After surgery, anesthesia was reversed with an intraperitoneal (IP) injection of atipamezol (0.5 mg/kg, ANTISEDAN®, Pfizer).

### Histology

At different time points after injection, animals were deeply anesthetized with an IP injection of pentobarbital (60 mg/kg, NEMBUTAL®, CEVA Santé Animale, Libourne, France) and transcardially perfused with phosphate buffered saline (PBS) followed by ice-cold 4% paraformaldehyde. After removal of the brain and overnight postfixation, 50 μm thick coronal sections were cut using a vibrating microtome (HM 650 V, Microm, Walldorf, Germany) and stored at 4 °C. Immunohistochemistry was performed under uniform conditions on free-floating sections using antibodies raised against eGFP (rabbit polyclonal 1:10000, in-house), TH (rabbit polyclonal 1:5000, Millipore AB152, Darmstadt, Germany), α-SYN (rabbit polyclonal 1:5000, Millipore AB5038) or P-S129 α-SYN (mouse monoclonal 1:5000, 11A5, Anderson *et al.* 2006 [34]; Elan Pharmaceuticals, South San Francisco, CA, USA). Sections were pretreated with 3% hydrogen peroxide for 10 min and incubated overnight with primary antibody in 10% normal goat or swine serum (DakoCytomation, Heverlee, Belgium). As secondary antibody, we used biotinylated anti-rabbit or anti-mouse IgG (1:600 (α-SYN), 1:300 (others), DakoCytomation), followed by incubation with streptavidin-horseradish peroxidase complex (1:1000, DakoCytomation). eGFP, α-SYN and P-S129 αSYN immunoreactivity was visualized using 3,3-diaminobenzidine (0,4 mg/ml, Sigma-Aldrich, St. Louis, MO, USA). TH immunoreactivity was visualized using Vector SG (SK-4700, Vector laboratories, Burlingame, CA) as a chromogen. After being rinsed and mounted, sections were coverslipped with DPX (Sigma-Aldrich).

For fluorescent double staining, sections were rinsed three times in PBS and then incubated overnight in PBS-0.1% triton X-100, 10% goat serum, and the following antibodies: TH (mouse monoclonal 1:500, Merck Millipore MAB318), eGFP (rabbit polyclonal 1:1000, in-house) and α-SYN (rabbit polyclonal 1:1000, Millipore AB5038). After three rinses in PBS-0.1% triton X-100 the sections were incubated in the dark for 2 h in fluorochrome-conjugated secondary antibodies: goat anti-rabbit Alexa 488 (1:500, Molecular Probes™, Invitrogen, Gent, Belgium) and goat anti-mouse Alexa 555 (1:500, Molecular Probes™, Invitrogen). After being rinsed in PBS and mounted, the sections were coverslipped with Mowiol. Fluorescent double staining for TH and eGFP or α-SYN was visualized by confocal laser scanning microscopy (Fluoview FV1000, Olympus, Tokyo, Japan) using a 40x lens.

### Stereological quantification

The total number of immunoreactive positive cells in the SN was estimated by stereological measurements using the optical fractionator method in a computerized system (StereoInvestigator; MicroBright-Field, Magdeburg, Germany) and a Leica DMR optical microscope as described before [60]. The SN pars compacta was delineated based on visual observation and morphology. Every fifth section throughout the rostro-caudal extent of the SN was analyzed, with a total of six sections for each animal. The coefficients of error, calculated according to the procedure of Schmitz and Hof as estimates of precision [61] varied between 0.07 and 0.16. The conditions of the experiment were blinded to the investigator. To determine the terminal density in the striatum pictures of TH stained sections were taken of 3 sections spaced 250 μm apart and analyzed using ImageJ. The number of P-S129 α-SYN-positive neuritic inclusions in the striatum was counted on a representative section 4 weeks after injection from the animals of the ‘high α-SYN dose’ experiment.

### Knockdown of parkin in SHSY5Y cells

To induce knockdown of parkin in cell culture different lentiviral vectors expressing a microRNA-based short-hairpin RNA (miR) against human parkin were generated as described [62]. The 2 target sequences resulting in the most potent knockdown on Q-RT-PCR (data not shown), miR1 parkin: CCAGAGGAAAGTCACCTGCGAA and miR2 parkin: ATGTAAAGAAGCGTACCATGAA, were used for further experiments. A miR against firefly luciferase is used as control [62]. Human dopaminergic SHSY5Y neuroblastoma cells stably overexpressing human WT α-SYN were transduced with lentiviral vectors expressing the miRs against parkin or control. Cells were maintained in DMEM (Invitrogen) supplemented with 15% fetal calf serum (Harlan Sera-Lab, International Medical), 1% non-essential amino acids (Invitrogen) and 50 μg/ml gentamycin (Invitrogen) and selected with blasticidin (10 μg/ml, Invitrogen). After selection cell extracts were generated in 1% SDS supplemented with protease cocktail inhibitor (Roche Diagnostics) and 8.5 μg of protein was loaded on gel. After electrophoresis and protein transfer the PVDF membranes (Bio-Rad) were blocked with 5% milk powder in PBS supplemented with 0.1% Tween 20. The membranes were incubated with the different primary antibodies against parkin (4211, 1/1000, Cell Signaling), P-S129 α-SYN (11A5, 1/2000), α-SYN (15G7, 1/1000, Enzo), protein phosphatase 2A (1/5000, kind gift of Dr. Veerle Janssens, Laboratory of Protein Phosphorylation and Proteomics, KU Leuven) and PLK2 (1/500, H-90, Santa Cruz). All samples were run on the same gel. Detection was performed after incubation with the appropriate HRP-conjugated secondary antibodies (Dako) using chemiluminescence (ECL^+^, Pierce).

### Statistical analysis

Statistical analysis was performed using GraphPad Prism 5.0 (GraphPad Software, La Jolla, CA, USA). Results are expressed as means ± standard error of the mean (SEM). Statistical analysis of the number of TH-positive cells, the number of Ser-129-P α-SYN positive cells and the number of α-SYN positive cells was carried out using two-way analysis of variance (ANOVA) followed by the Bonferroni *post-hoc* test for intergroup comparisons. To compare the number of P-S129 α-SYN positive cells at the different time points between the two genotypes in the experiment with the low titer of rAAV2/7-WT α-SYN, a Student’s *T*-test was performed and P-values were adjusted with the Benjamini-Hochberg procedure, with the false discovery rate set at 0.05, to avoid accumulation of α-errors in multiple T-tests.

Statistical significance level was set as follows: * if P < 0.05, ** if P < 0.01, *** if P < 0.001.

## Abbreviations

ANOVA: Analysis of variance
α-SYN: Alpha-synuclein
eGFP: Enhanced green fluorescent protein
GC: Genome copies
IP: Intraperitoneal
LBs: Lewy bodies
PD: Parkinson’s disease
parkin^−/−^: parkin knockout
parkin^+/+^: parkin wild type
PBS: Phosphate buffered saline
P-S129 α-SYN: α-SYN phosphorylated at serine residue 129
rAAV: recombinant adeno-associated viral vector
SEM: Standard error of the mean
SN: Substantia nigra
TH: Tyrosine hydroxylase
WT: Wild type
